# Identification and Characterization of Csa-miR395s Reveal Their Involvements in Fruit Expansion and Abiotic Stresses in Cucumber

**DOI:** 10.3389/fpls.2022.907364

**Published:** 2022-06-15

**Authors:** Lin Lu, Weirong Luo, Wenjin Yu, Junguo Zhou, Xinfa Wang, Yongdong Sun

**Affiliations:** ^1^School of Horticulture and Landscape Architecture, Henan Institute of Science and Technology, Xinxiang, China; ^2^Henan Province Engineering Research Center of Horticultural Plant Resource Utilization and Germplasm Enhancement, Xinxiang, China; ^3^College of Agriculture, Guangxi University, Nanning, China

**Keywords:** cucumber, miR395, gene expression, fruit expansion, abiotic stress

## Abstract

The miR395 plays an indispensable role in biochemical processes by regulating their target genes. However, little is known about the roles of miR395 in cucumber fruit expansion and response to abiotic stresses. Here, 4 Csa-miR395s and 8 corresponding target genes were identified in the cucumber genome. Csa-miR395s were all located on the same chromosome (Chr 5). Csa-miR395a/b/c and Csa-miR395d were distributed in different branches without a closer genetic relationship. Massive cis-acting elements, including light, phytohormone, and stress response elements, were detected in the promoter regions of Csa-MIR395s, indicating that Csa-miR395s might be involved in complex regulatory networks to control cucumber growth and development and stress response. In addition, Csa-miR395a/b/c shared the same target genes, and Csa-miR395d had its specific target genes. Tissue-specific expression analysis showed that Csa-miR395a/b/c were all expressed in the leaf, root, ovary, and expanded fruit of cucumber and highly expressed in the expanded fruits compared to the ovary, while Csa2G215520 and Csa1G502860 (target genes of Csa-miR395a/b/c) presented a downregulated trend in the expanded fruit compared to the ovary. Meanwhile, the protein co-expression network revealed that these target genes had interactions in sulfur metabolism. These results suggested that Csa-miR395a/b/c targeting Csa2G215520 and Csa1G502860 might promote cucumber fruit expansion by affecting sulfur metabolism. Additionally, Quantitative Real-time PCR analysis validated that Csa-miR395s could be regulated by NaCl stress, and Csa-miR395a/b/c could respond to PEG stress, which further confirmed the reliability of cis-acting elements data. Taken together, our results could be helpful for further exploration of the functions of miR395s in cucumber fruit expansion and response to abiotic stresses.

## Introduction

Cucumber (*Cucumis sativus* L.) is one of the most important horticultural crops, which is widely cultivated around the world and with important economic and social value. In 2020, the cultivated area of cucumber in China reached 1.27 million ha, accounting for 56.4% of the global total area. Also, 90.35 million tons of cucumber were produced in the world, and China accounted for 73.36 million tons, accounting for 81.2% of the global production (Food Agriculture Organization of the United Nations, 2021). Fruit expansion is a complicated biological process, which directly influences the yield and quality of cucumber, and is frequently suppressed by various biotic and abiotic stresses. It is very necessary to elucidate the molecular mechanisms of cucumber fruit expansion and protect it from adverse conditions for higher yield and quality.

The microRNA (miRNA) is a kind of endogenous non-coding small RNA with a length of 21–25 nucleotides, which is ubiquitous in organisms (Jones-Rhoades and Bartel, 2004). It mainly regulates gene expression at the post-transcriptional level through complementing and pairing with their target genes, resulting in mRNA degradation or inhibition of translation (Jones-Rhoades et al., 2006), and then, participates in a series of biochemical reactions, such as plant growth and development, metabolism, stress response, and so on (Zhang et al., 2006; Ferdous et al., 2015; Teotia and Tang, 2015). In addition, miRNA has also been reported to be the regulator of fruit development processes involved in fruit coloration, fruit initiation, and fruit ripening (Zhang et al., 2017; Chen et al., 2018). It was hypothesized that miRNA would regulate cucumber fruit expansion. Therefore, it will help resolve the problem of fruit production to recognize the miRNA associated with fruit expansion, as well as discover the potential functions. In the previous study, we identified miR395 from the expanded cucumber fruit small RNA library (Sun et al., 2019).

The miR395 is one of the highly conserved miRNAs during evolution (Yuan et al., 2016) and participates in plant metabolism *via* regulating downstream target genes. In a general sense, the target genes of miR395 are divided into two main categories, namely, sulfate transporters and ATP sulfurylase (adenosine sulfate acyltransferase, ATPS) (Jones-Rhoades and Bartel, 2004; Ferdous et al., 2015; Yuan et al., 2016). Sulfate transporters mediate the transmembrane transport of sulfate and participate in the uptake and distribution of sulfate in plant cells (Takahashi, 2010; Takahashi et al., 2011b). ATPS is the first key enzyme involved in sulfate activation and assimilation (Hatzfeld et al., 2000; Ai et al., 2016). After inorganic sulfate from the soil has been ingested, it must first be catalyzed by ATPS to form adenosine 5′-phosphosulfate (APS), and then, go through a series of assimilation processes to form cysteine that can be directly incorporated into proteins or peptides, such as glutathione (GSH), which plays important roles in storage and transport of sulfate and stress defense in plants (Rotte and Leustek, 2000; Vauclare et al., 2002). Previously, it has been reported that miR395 regulates its target genes to affect the absorption and assimilation of sulfate in plants and then, influences a series of biosynthesis and development processes (Buchner et al., 2004; Ai et al., 2016). Liang et al. (2010) revealed that miR395 regulated the accumulation of sulfate in the shoot by targeting *APS* genes and the redistribution of sulfate between leaves by cleaving *SULTR2;1* in *Arabidopsis*. The miR395-*APS3* module has been reported to be directly involved in the development of Chinese kale seeds and the sulfur metabolism during seed development (Tang et al., 2022). The upregulated expression of miR395a and the downregulated expression of *APS1* might promote the morphogenesis of spherical embryos in *Dimocarpus longan* (Huang et al., 2020). Additionally, miR395 with an upregulated expression might play a regulatory role in response to NaCl stress in *Halostachys caspica* (Yasin, 2014). Upregulated expression of miR395 could ease the damage from low temperature in cassava (Zeng et al., 2014) and *Musa itinerans* (Liu, 2018). Pre-MIR395d of *Arabidopsis* improved the detoxification ability of heavy metal cadmium in *Brassica napus* (Zhang et al., 2013). These results indicated that miR395 could be involved in response to abiotic stresses in a variety of plants, while, there were few reports on Csa-miR395s regulation in cucumber fruit expansion and response to abiotic stresses.

To gain insight into miR395s and elucidate their potential functions in cucumber, four Csa-miR395s and eight corresponding target genes in cucumber were identified and characterized through bioinformatics analysis in this current study. Furthermore, expression patterns of Csa-miR395s and their target genes in different tissues were visualized using Quantitative Real-Time PCR (qRT-PCR) analysis and RNA-seq data. Lastly, expression profiles of Csa-miR395s under PEG and NaCl stresses were investigated to provide a reference for the possible functions in response to abiotic stresses. Our results will provide valuable information for further functional analysis of Csa-miR395s in cucumber fruit expansion and response to abiotic stresses.

## Materials and Methods

### Identification of Csa-miR395s and Their Potential Target Genes

The miR395s in cucumber were searched through the online website Ensembl Plants (http://plants.ensembl.org/). Taking the cucumber transcriptome database as the potential target gene database and the mature sequences of Csa-miR395s as the object, the target genes of Csa-miR395s were successfully predicted *via* psRNA-Target software (https://www.zhaolab.org/psRNATarget/analysis). The genes with values below 2.5 were selected as the target genes of Csa-miR395s.

### The Chromosomal Localization of Csa-miR395s and Prediction of Secondary Structure

The chromosomal localization of Csa-miR395s was mapped using the MapGene2Chromosome V2.1 program (http://mg2c.iask.in/mg2c_v2.1/). The precursor sequences of Csa-MIR395s were retrieved from the Ensembl Plants database. Under default parameters, the secondary structures, and the lowest folding free energy of pre-MIR395s were predicted using the RNA secondary structure prediction website (http://rna.urmc.rochester.edu/RNAstructureWeb/Servers/Predict1/Predict1.html).

### Construction of Phylogenetic Tree

The mature sequences of miR395s from eight different species, including cucumber, melon, *Arabidopsis*, rice, tobacco, watermelon, pumpkin, and tomato, were retrieved from miRBase (http://www.mirbase.org/) and PmiREN databases (https://www.pmiren.com/). Multiple sequence alignment on these mature sequences was implemented using ClustalW software, and the phylogenetic tree was constructed *via* MEGA V11.0 software, with the neighbor-joining (NJ) method. The bootstrap value was set to 1,000, and other parameters were default.

### Analysis of Cis-Acting Elements in the Promoter Regions of Csa-miR395s

The promoter sequences of Csa-MIR395s, at least 2 kb of the upstream regions, were downloaded from the Ensembl Plants database. Then, the cis-acting elements in the promoter regions of Csa-MIR395s were analyzed using the PlantCARE database (http://bioinformatics.psb.ugent.be/webtools/plantcare/html/). Finally, it was visualized by an Excel chart.

### Plant Growth Condition, Stress Treatment, and Sample Preparation

Cucumber seeds (Jinyou No. 1) were cultivated in a glass culture dish at 28°C and induced to germinate after they were soaked at 55°C for 15–20 min. The germinated seeds were sown in a pot (peat soil: perlite: vermiculite = 2:1:1) and then, placed in a laboratory climate box (light for 16 h, darkness for 8 h, 28°C/18°C). When the cotyledons of these seedlings were expanded, they were transferred to the plastic greenhouse of Henan Institute of Science and Technology (Xinxiang, China) for continuous growth. Tissue samples of young root, leaf, ovary (on the day of anthesis), and expanded fruit (5 days post-anthesis) were collected from cucumber plants with the same growth.

For abiotic stress treatments, the cucumber seedlings at the two-leaf stage were submerged in a nutrient solution, containing 10% PEG6000 or 150 mmol/L NaCl to stimulate drought or salt stress, respectively. Leaf tissue was collected at 0, 3, 6, 12, and 24 h after treatment. All treatments were repeated three times, with at least three cucumber seedlings treated each time.

### Expression Patterns of the Target Genes of Csa-miR395s

The National Center for Biotechnology Information (NCBI) SRA database (https://www.ncbi.nlm.nih.gov/sra/) was used to obtain RNA-seq data on cucumbers. Then, the RNA-seq data (PRJNA80169) were utilized to analyze the expression patterns of target genes of Csa-miR395s in four tissue samples (root, leaf, ovary and expanded fruit) of cucumber. The transcript levels of these target genes were computed by the reads per kilobase of exon per million reads mapped (RPKM) method. The obtained RPKM values of target genes were Log2 normalized. The gene expression heatmap was visualized based on RPKM values through TBtools software.

### Interaction Network of the Target Genes of Csa-miR395s

Multiple protein sequences of these target genes were submitted to the STRING database (https://string-db.org/) with *Arabidopsis thaliana* as a reference organism, and the genes with the highest bit score were used to construct protein interaction networks.

### qRT-PCR Analysis of Csa-miR395s

RNA of these cucumber samples was extracted using TaKaRa MiniBEST Plant RNA Extraction Kit (TaKaRa, Dalian, China). After agarose gel electrophoresis and detecting the concentration by NanoDrop One, qualified RNA samples were acquired. Then, the qualified RNA was used for reverse transcription. First-strand cDNA was synthesized using Mir-X miRNA First-Strand Synthesis Kit (TaKaRa, Dalian, China), with a modified oligo (dT) primer and SMART MMLV Reverse Transcriptase, according to the manufacturer's protocol. The qRT-PCR experiment was implemented with TB Green® Premix Ex Taq™ II (Tli RnaseH Plus) (TaKaRa, Dalian, China) regarding U6 snRNA as the reference gene for Csa-miR395s (Wen et al., 2020). Primer sequences for Csa-miR395s are listed in Supplementary Table S1. All operations were carried out by the instructions. The relative expression levels of Csa-miR395s were analyzed using the 2^−ΔΔCt^ algorithm (Livak and Schmittgen, 2001), repeated three times for each experiment.

## Results

### Identification and Secondary Structure of Csa-miR395s

**Figure 1:**
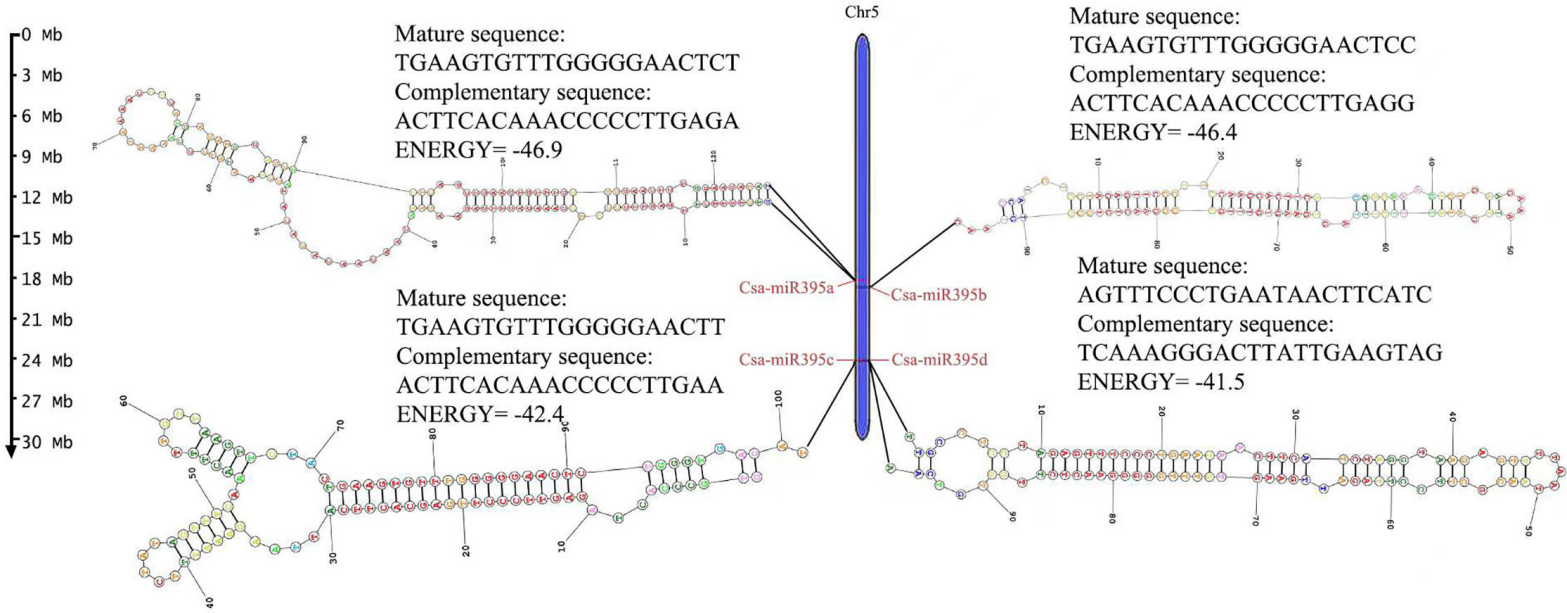
Chromosome localization of Csa-miR395s. The scale was in megabases (Mb). The secondary structure and mature sequences of Csa-MIR395s were provided.

Four miR395s were identified in cucumber. The length of the precursor sequences of Csa-MIR395s ranged from 95 to 126 nt, and mature sequences ranged from 20 to 22 nt, respectively. Four Csa-miR395s were all distributed on the same chromosome (Chr 5) in cucumber (Figure 1) and named Csa-miR395a, Csa-miR395b, Csa-miR395c, and Csa-miR395d, respectively, according to the position. The mature sequences of Csa-miR395a/b/c were highly consistent, while the mature sequence of Csa-miR395d was different from those of Csa-miR395a/b/c, although they were located on the same chromosome.

The secondary structure of pre-MIR395 sequences was predicted by an RNA structure online website. It was found that four pre-MIR395 sequences all formed a typical stem-loop structure. The lowest folding free energy of pre-MIR395s ranged from −46.9 Kal/mol (pre-MIR395a) to −41.5 Kal/mol (pre-MIR395d) (Figure 1). The number of sub-loops varied from 5 (pre-MIR395c) to 7 (pre-MIR395a/d).

### Evolutionary Relationship of miR395s

**Figure 2:**
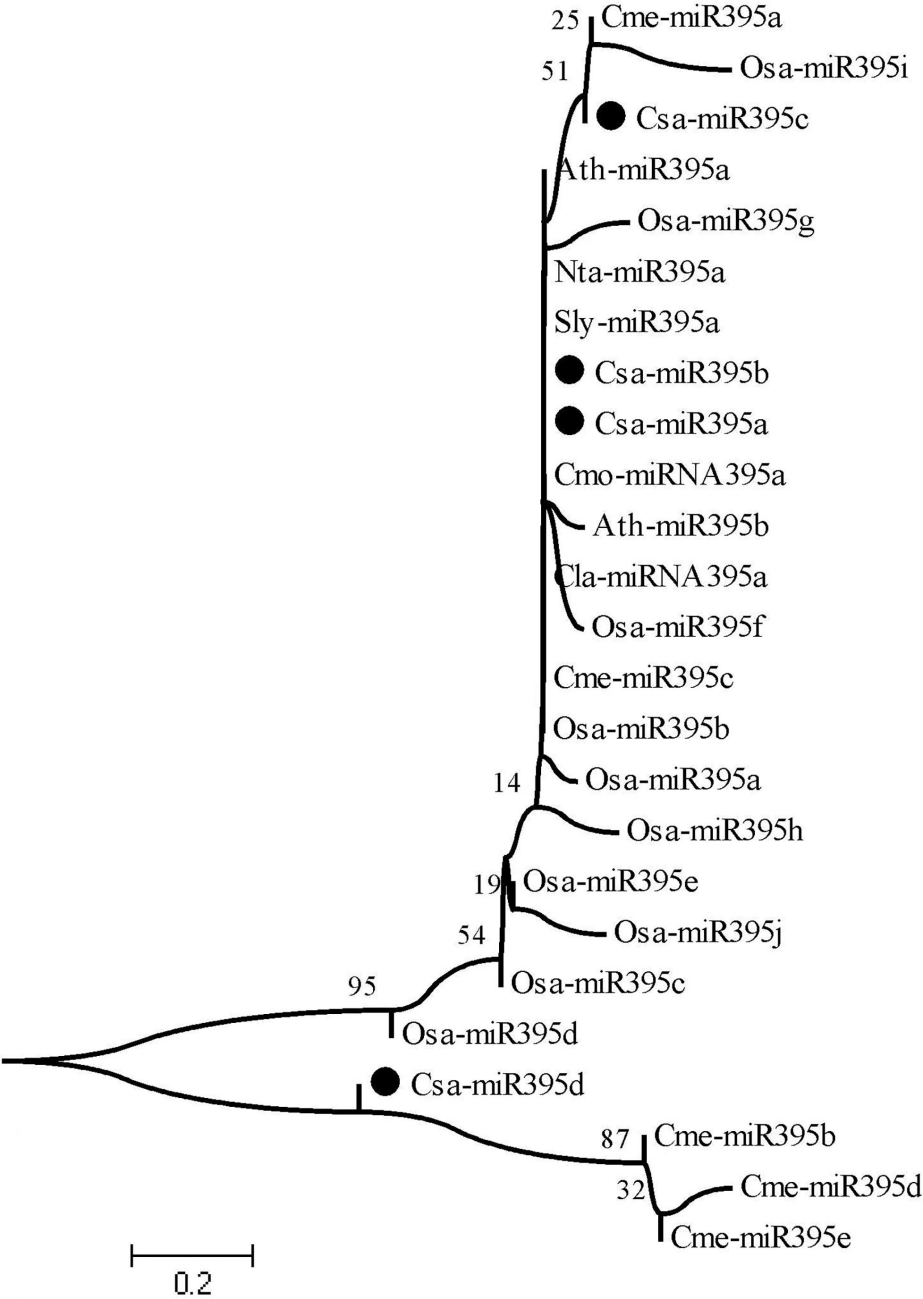
Evolutionary relationship of miR395s. Csa, cucumber, Cme, melon, Ath, *Arabidopsis*, Osa, rice, Sly, tomato, Nta, tobacco, Cla, watermelon, Cmo, pumpkin.

To explore the evolutionary relationship of miR395s between cucumber and other species, mature sequences of eight different species, including cucumber (Csa-miR395a-d), melon (Cme-miR395a-e), *Arabidopsis* (Ath-miR395a/b), rice (Osa-miR395a-j), tobacco (Nta-miR395a), watermelon (Cla-miR395a), pumpkin (Cmo-miR395a), and tomato (Sly-miR395a) were used to construct the phylogenetic evolution tree using MEGA v11.0 (Figure 2). As a result, 25 of miR395 members were clustered into two branches. Csa-miR395d and Cme-miR395b/d/e belonged to one branch. Csa-miR395a/b/c were classified into another branch with remaining members. Furthermore, the evolutionary processes of Csa-miR395a/b/c were more similar to one another than those with Csa-miR395d, perhaps because of high sequence similarities, while Csa-miR395d shared a closer genetic relationship with Cme-miR395b/d/e in melon.

### Cis-Acting Elements of Csa-miR395s

**Figure 3:**
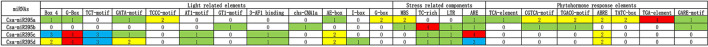
Cis-acting elements in the promoter regions of Csa-MIR395s. These cis-acting elements were divided into three main categories; light-related elements, stress-related components, and phytohormone-response elements. The number of these cis-acting elements was represented by different colors.

To better understand the functions of Csa-miR395s, the cis-acting elements of Csa-MIR395s were analyzed *via* the PlantCARE database (Figure 3). The results showed that the cis-acting elements of Csa-MIR395s could be classified into three main categories, including light (Box 4, 3-AF1 binding, chs-CMA1a, G-Box, AE-box, I-box, G-box, TCT-motif, GATA-motif, TCCC-motif, AT1-motif, and GT1-motif), stress (MBS, TC-rich, LTR, and ARE), and phytohormone (ABRE, TATC-box, TCA-element, TGA-element, CGTCA-motif, TGACG-motif, and GARE-motif) response elements. Two MBSs (an MYB-binding site involved in drought inducibility), 1 ARE (involved in anaerobic induction), 2 CGTCA-motifs (involved in the MeJA responsiveness), 2 TGACG-motifs (MeJA responsiveness), 1 TCA element (involved in salicylic acid responsiveness), and 2 ABREs (involved in the abscisic acid responsiveness) were discovered in the promoter region of Csa-MIR395a. One MBS, 1 ARE, 1 CGTCA-motif, and 1 TGACG-motif were identified in Csa-MIR395b. Four AREs and 2 ABREs were found in Csa-MIR395c. Also, 3 AREs and 2 ABREs were detected in Csa-MIR395d.

### Target Genes of Csa-miR395s

To further understand the regulatory networks of Csa-miR395s, we predicted eight potential target genes of Csa-miR395s using the online website psRNA-Target. The results showed that Csa-miR395a/b/c shared the same target genes (Csa4G031010, Csa2G215520, Csa3G144230, Csa5G407060, Csa1G502860, and Csa3G738990), while Csa-miR395d had its own specific target genes (Csa2G070900 and Csa2G357320) (Table 1). Csa4G031010 and Csa5G407060 were sulfate transporter 2.1 and sulfate transporter 2.2, respectively. Csa2G215520 was *ATPS4*. Csa1G502860 was nodulin homeobox. Csa3G144230, Csa3G738990, Csa2G070900, and Csa2G357320 were all hypothetical protein.

**Table 1:** Target genes of Csa-miR395s.

**Name**	**Target Acc**.	**Gene ID**	**Except**	**Inhibition**	**Description**
Csa-miR395a/b/c	KGN53230	Csa4G031010	0.0	Cleavage	Sulfate transporter 2.1
	KGN61658	Csa2G215520	1.0	Cleavage	ATP sulfurylase 4, chloroplastic
	KGN56933	Csa3G144230	1.0	Cleavage	Hypothetical protein
	KGN51012	Csa5G407060	1.5	Cleavage	Sulfate transporter 2.2
	KGN65698	Csa1G502860	2.5	Cleavage	Nodulin homeobox
	KGN58953	Csa3G738990	2.5	Cleavage	Hypothetical protein
Csa-miR395d	KGN61224	Csa2G070900	2.5	Cleavage	Hypothetical protein
	KGN62509	Csa2G357320	2.5	Cleavage	Hypothetical protein

### Expression of Csa-miR395s in the Different Tissues

**Figure 4:**
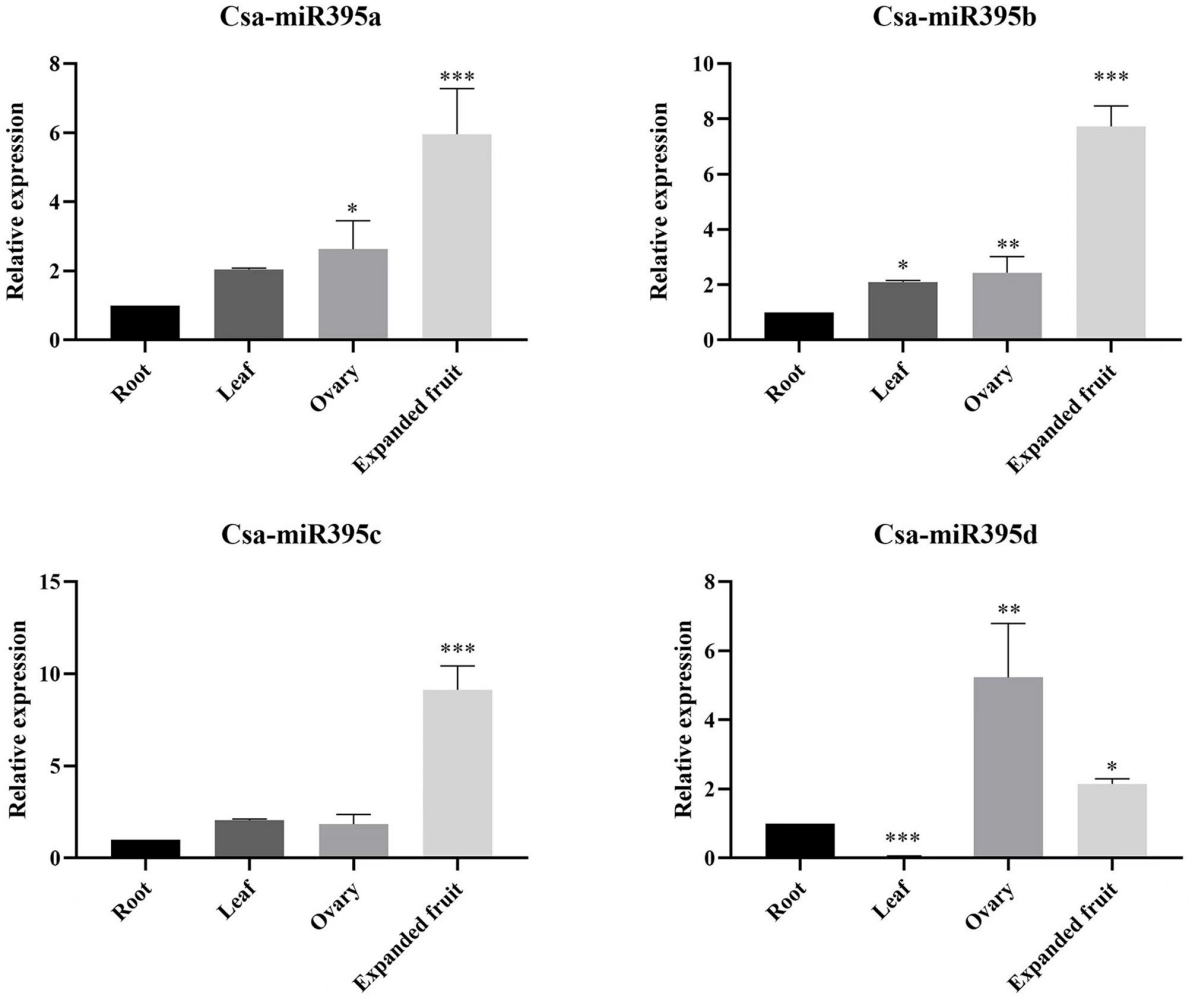
Expression patterns of Csa-miR395s in the different tissues (root, leaf, ovary, and expanded fruit). The X-axis represents the tested tissue samples. The Y-axis represents the relative expression level of a transcription factor. The values represent the means ± SDs. Asterisks represent significant difference at *p* ≤ 0.05 (*), *p* ≤ 0.01 (**), and *p* ≤ 0.001 (***).

To confirm the potential functions of Csa-miR395s in cucumber growth and development, the expression profiles of Csa-miR395s were detected by qRT-PCR in the root, leaf, ovary, and expanded fruit of cucumber (Figure 4). The results showed that the expression patterns of Csa-miR395a/b/c are consistent, and Csa-miR395a/b/c exhibited the highest expression levels in expanded fruit and the lowest levels in root. Compared to the ovary, the expression levels of Csa-miR395a/b/c were upregulated by 2.27-fold, 3.17-fold, and 4.98-fold in expanded fruit, respectively, while the expression level of Csa-miR395d in the expanded fruit was lower than that in the ovary. Our findings indicated that Csa-miR395a/b/c might play an active role in regulating cucumber fruit expansion, especially from the ovary to the expanded fruit.

### Expression of the Target Genes in the Different Tissues

**Figure 5:**
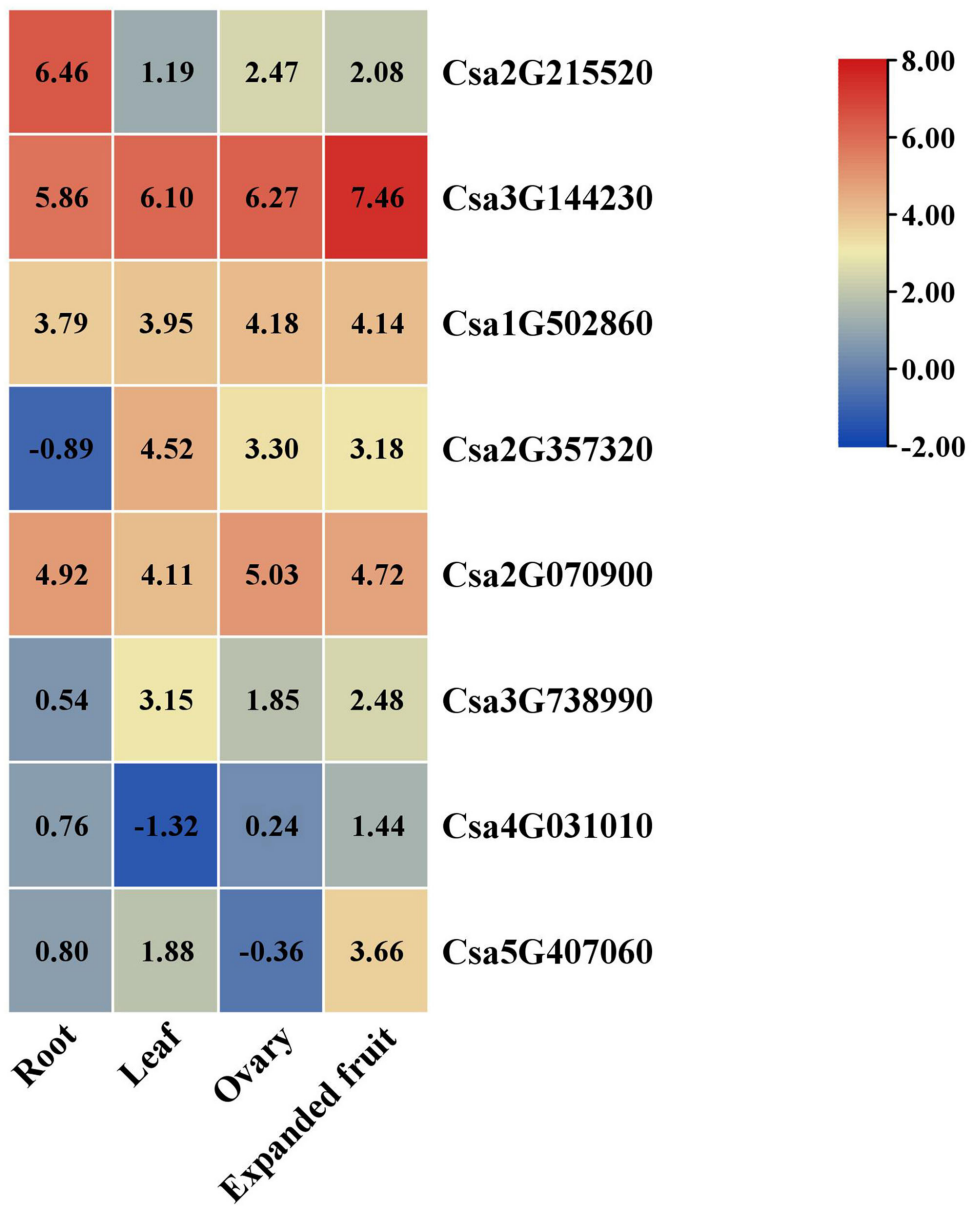
Expression patterns of the target genes in the different tissues (root, leaf, ovary, and expanded fruit). The reads per kilobase of exon per million reads mapped (RPKM) values of these target genes were performed by log2. Differences in gene expression were shown by color. The red and blue colors represent the higher and lower relative expressions of the transcript, respectively.

Based on RNA-seq data, the expression patterns of the target genes of Csa-miR395s in four different tissues (root, leaf, ovary, and expanded fruit) were visualized to confirm the potential relationships between Csa-miR395s and their target genes (Figure 5). The results showed that the expression patterns of Csa2G215520 in the root, ovary and expanded fruit were negatively correlated with those of Csa-miR395a/b/c, suggesting that Csa2G215520 was the major target gene of Csa-miR395a/b/c. Compared to the ovary, the target genes (Csa2G215520 and Csa1G502860) of Csa-miR395a/b/c showed a downregulated trend in the expanded fruit. These results were negatively correlated with those of Csa-miR395a/b/c, while, the transcript abundances of the target genes (Csa2G070900 and Csa2G357320) for Csa-miR395d in the expanded fruit were lower than those in the ovary, which were consistent with that of Csa-miR395d. Based on these results, we speculated that Csa-miR359a/b/c targeted Csa2G215520 and Csa1G502860 to regulate cucumber fruit expansion.

### Interaction Network Analysis of the Target Genes

**Figure 6:**
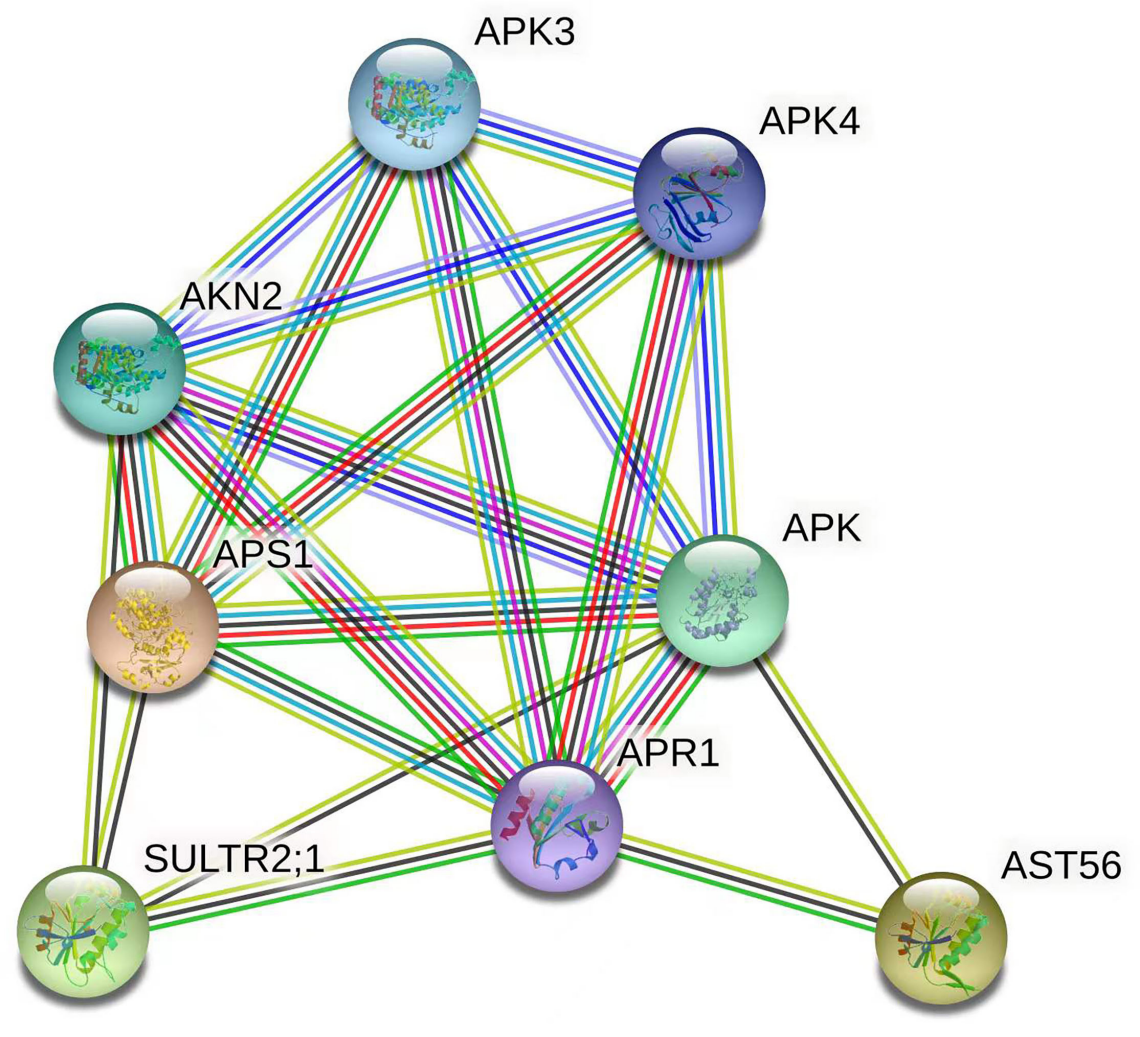
Interaction network of the target genes of Csa-miR395a/b/c. Network nodes represent proteins. Edges represent protein-protein associations. The 3D protein structure was displayed inside the nodes.

Given the qRT-PCR results and RNA-seq data, the interaction network of the target genes of Csa-miR395a/b/c was constructed to explore the relationships of these target genes and better understand the regulation mechanism of Csa-miR395a/b/c in cucumber fruit expansion. It could be seen from Figure 6 that Csa2G215520 (APS1) shared interaction relationships with Csa4G731010 (SULTR2;1), Csa5G407060 (AST56), and Csa3G144230 (APS1) using *Arabidopsis thaliana* as reference species, which affected sulfate absorption and assimilation in plants. The genes (Csa3G738990 and Csa1G502860) unassociated with the protein network were not shown. To explore the relationships of these target genes in more detail, five predicted functional partners (APR1, APK, APK3, APK4, and AKN2) were added. In plants, APS was utilized by APR, APK, and AKN to enter two different sulfate metabolism pathways (Leustek et al., 2000). Therefore, we speculated that Csa-miR395a/b/c regulated sulfur assimilation through the protein-protein interaction network among its target genes, and then, played roles in the regulation of plant fruit expansion.

### Expression Patterns of Csa-miR395s in Response to Abiotic Stress

**Figure 7:**
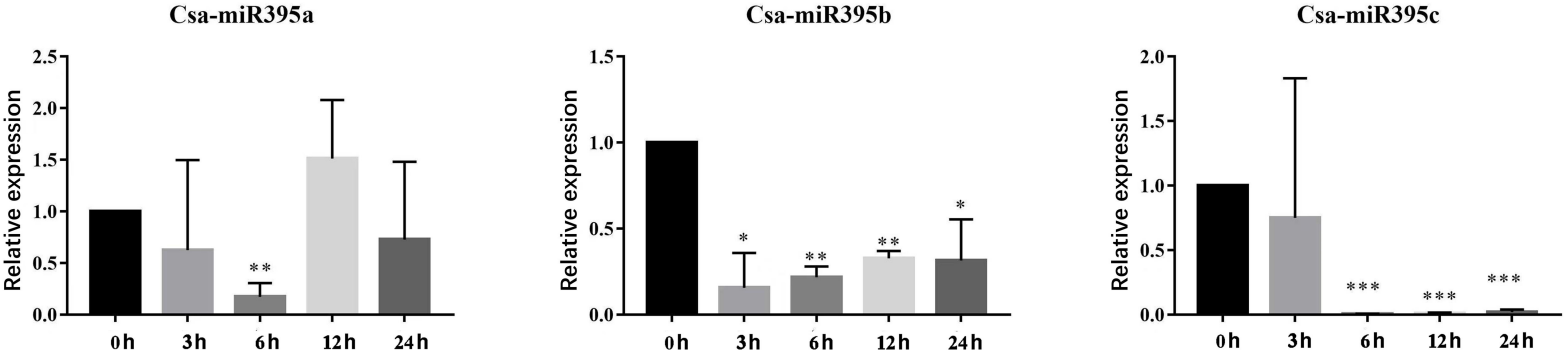
Expression levels of Csa-miR395s in response to PEG stress (0, 3, 6, 12, and 24 h). The X-axis represents the hours of 10% PEG6000 treatment. The Y-axis represents the relative expression level of a transcription factor. The values represent the means ± SDs. The significant difference is represented by asterisks according to *t*-test (*p < 0.05, **p < 0.01, and ***p < 0.001).

**Figure 8:**
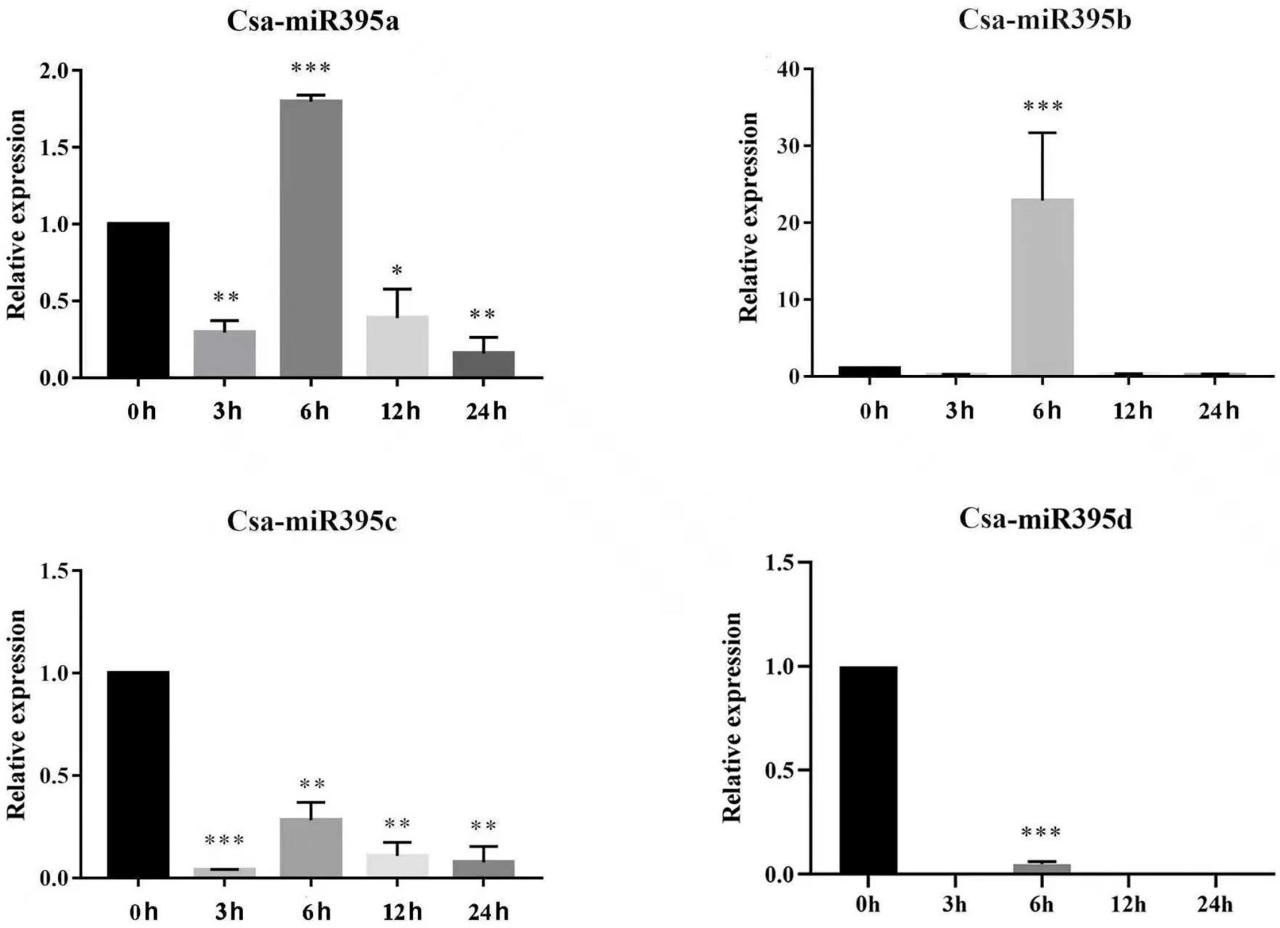
Expression levels of Csa-miR395s in response to NaCl stress (0, 3, 6, 12, and 24 h). The X-axis represents the hours of 150 mmol/L NaCl treatment. The Y-axis represents the relative expression level of a transcription factor. The values represent the means ± SDs. The significant difference is represented by asterisks according to *t*-test (*p < 0.05, **p < 0.01, and ***p < 0.001).

miR395 has been confirmed to be involved in response to abiotic stresses among a variety of plants (Li et al., 2017; Fu et al., 2019). To investigate the potential functions of Csa-miR395s under abiotic stresses, their transcript abundances were performed under PEG and NaCl treatments by qRT-PCR. Under PEG treatments (Figure 7), Csa-miR395a was downregulated at 3-/6-/24-h time points, while upregulated at a 12-h time point in contrast with the normal condition. Csa-miR395b/c were both downregulated at all time points, and significantly downregulated at 6-/12-/24-h time points. The transcription level of Csa-miR395d was undetectable in PEG treatments. Under NaCl treatments (Figure 8), the expression levels of Csa-miR395a/b/c were downregulated at 3-/12-/24-h time points, and Csa-miR395a/c showed the significant downregulation, compared with the normal condition. Csa-miR395a/b showed remarkably upregulated expression at a 6-h time point. Meanwhile, Csa-miR395d was only expressed at a 6-h time point, and the expression level was significantly downregulated in contrast with the normal condition. Thus, our results confirmed that Csa-miR395s could be regulated by NaCl stress, and Csa-miR395a/b/c could respond to PEG stress, suggesting that Csa-miR395s might be involved in plant response to abiotic stresses.

## Discussion

The miR395 has been characterized in numerous species, such as *Arabidopsis* (Liang et al., 2010), *Halostachys caspica* (Yasin, 2014), cassava (Zeng et al., 2014), *Musa itinerans* (Liu, 2018), and *Brassica napus* (Zhang et al., 2013). However, the characteristic and the function of miR395 in cucumber have rarely been reported. In the present study, we identified four miR395s in cucumber from the Ensembl Plants database and successfully predicted their target genes, and characterized them by investigating their chromosomal distribution, evolutionary relationship, cis-acting elements, gene expression profiles under different tissues, and abiotic stresses. Our results will provide directions for further study on Csa-miR395s regulation in cucumber fruit expansion and response to abiotic stresses.

Sequence data showed that the mature sequences of Csa-miR395a/b/c were highly homologous, and there were obvious differences between Csa-miR395d and Csa-miR395a/b/c, while Csa-miR395d shared the same chromosome with Csa-miR395a/b/c. By analyzing the evolutionary relationship of miR395s from eight species, we found that the number of miR395 was different in different species, which might be due to the complexity of the evolutionary process. In addition, we also found that four Csa-miR395s were located on the same chromosome, while the evolutionary processes of Csa-miR395a/b/c and Csa-miR395d were distributed in different branches without a closer genetic relationship because of the differential mature sequences, suggesting that it was not a simple tandem duplication event. A similar observation was reported for Csa-miR160s in cucumber (Li et al., 2022).

The miRNA participates in the whole process of plant growth and development by regulating the expression of its target genes. In this work, eight target genes of Csa-miR395s were predicted by psRNA-Target software. Csa-miR395a/b/c possessed the same target genes, *ATPS*, and sulfate transporters, while Csa-miR395d had its specific target genes. The *ATPS* and sulfate transporters have been reported to promote the uptake and assimilation of sulfate in plants (Buchner et al., 2004; Takahashi, 2010). The protein co-expression network also verified that these target genes had interactions in sulfur metabolism. Besides, the effects of miRNA on sulfur assimilation have been previously reported (Takahashi et al., 2011a; Faraji et al., 2021). It is possible that miR395 regulates fruit expansion by affecting nutrient uptake. However, more research is needed.

Tissue-specific expression is usually performed to predict the gene functions during plant growth and development. Here, we used four different tissues to explore the expression profiles of Csa-miR395s and their target genes in cucumber. qRT-PCR analysis revealed that Csa-miR395a/b/c were all expressed in the four tissues and exhibited different but partially overlapping expression patterns. Compared to the ovary, the transcription levels of Csa-miR395a/b/c were significantly upregulated in the expanded fruit, indicating that Csa-miR395a/b/c might be the active regulator of cucumber fruit expansion. Similarity, miR395 showed a similar expression pattern in *Dimocarpus longan* with the upregulated expression trend at the early stage of somatic embryogenesis (Huang et al., 2020). miRNA regulates the process of plant growth and development by inhibiting the expression of its target genes. According to the expression patterns of Csa-miR395s and their target genes in different tissues, Csa2G215520 was the major target gene of Csa-miR395a/b/c. Compared with the ovary, the transcript abundances of Csa2G215520 and Csa1G502860 (target genes of Csa-miR395a/b/c) were downregulated in the expanded fruit, which was negatively correlated with those of Csa-miR395a/b/c. Thus, Csa-miR395a/b/c targeting Csa2G215520 and Csa1G502860 might promote cucumber fruit expansion by affecting sulfur metabolism.

There were an increasing number of reports that miR395 might play fundamental regulatory roles in response to abiotic stresses, including sulfate deficiency (Liang et al., 2010), heavy metal cadmium (Zhang et al., 2013; Fu et al., 2019), low temperature (Zeng et al., 2014), blu-ray (Li et al., 2018), and salinity (Yasin, 2014). In the current study, a lot of cis-acting elements were discovered in the promoter regions of Csa-MIR395s, and these regulation components could be classified into three main categories, including light, phytohormone, and stress-response elements. It has been reported that these three phytohormone response elements, such as abscisic acid (ABRE, ACGTG), salicylic acid (TCA-element), and methyl jasmonate (CGTCA-motif and TGACG-motif), can be involved in regulating abiotic stresses through the plant signal transduction pathway (Doornbos et al., 2011; Rivas-San Vicente and Plasencia, 2011). Here, abscisic acid, salicylic acid, and methyl jasmonate response elements were detected in the promoter regions of Csa-MIR395s. Meanwhile, MBS and ARE were highly enriched in the promoters of Csa-MIR395s. This indicated that Csa-miR395s might be involved in the signal transduction pathways of various stresses. Consistent with this, Csa-miR395s could be induced by NaCl stress, and Csa-miR395a/b/c could respond to PEG stress based on quantitative expression analysis. For example, Csa-miR395a/b/c showed a downregulated expression trend under PEG treatments. Similar findings of miR395 were also reported in *Oryza sativa* (Zhou et al., 2010) and mulberry (Han, 2015). Moreover, the expression levels of Csa-miR395s showed a trend of decreasing and then increasing, and 6 h was the main response time point in response to NaCl treatments. It suggested that, when cucumber was stimulated by NaCl stress, it first adapted to the environment through self-protection, and then regulated its adaptability through NaCl stress-related resistance genes. Csa-miR395s might be the main NaCl stress resistance genes, and 6 h might be the main response time point to NaCl stress.

## Conclusions

In this study, 4 Csa-miR395s and their 8 corresponding target genes were identified in cucumber. A comprehensive analysis was performed, including bioinformatics and expression profiles, to discover the potential functions of Csa-miR395s. The results showed that Csa-miR395s were located on the same chromosome, while the evolutionary processes of Csa-miR395a/b/c and Csa-miR395d were distributed in different branches without a closer genetic relationship. Csa-MIR395s owned various kinds of cis-acting elements in their promoters, implying that Csa-miR395s might have crucial roles in regulating cucumber growth and development and stress response. The qRT-PCR analysis validated that Csa-miR395s were involved in response to NaCl stress, and Csa-miR395a/b/c could be regulated by PEG stress. Furthermore, qRT-PCR, RNA-seq data, and protein co-expression network showed that Csa-miR395a/b/c targeting Csa2G215520 and Csa1G502860 might promote cucumber fruit expansion by affecting sulfur metabolism. As a whole, our results will lay a foundation for further exploration of the functions of miR395s in cucumber fruit expansion and response to abiotic stresses.

## Data Availability Statement

The datasets presented in this study can be found in online repositories. The names of the repository/repositories and accession number(s) can be found below: https://www.ncbi.nlm.nih.gov/bioproject/PRJNA80169.

## Author Contributions

YS conceived and designed the experiments. LL carried out the experiments and wrote the manuscript. LL, WL, JZ, and XW analyzed the data and prepared figures and tables. YS and WY reviewed the manuscript. All the authors have read and approved the final manuscript.

## Funding

This research was funded by the Key Research and Development Program of Henan Province (No. 202102110040; No. 212102110130) and the Key Science and Technology Program of Xinxiang City (No. GG2019013).

## Conflict of Interest

The authors declare that the research was conducted in the absence of any commercial or financial relationships that could be construed as a potential conflict of interest.

## Publisher's Note

All claims expressed in this article are solely those of the authors and do not necessarily represent those of their affiliated organizations, or those of the publisher, the editors and the reviewers. Any product that may be evaluated in this article, or claim that may be made by its manufacturer, is not guaranteed or endorsed by the publisher.
