# Supplementary material for: Identification and Characterization of Csa-miR395s Reveal Their Involvements in Fruit Expansion and Abiotic Stresses in Cucumber
Source: Front Plant Sci. 2022 Jun 15;13:907364. doi: 10.3389/fpls.2022.907364 (PMC9240705; doi:10.3389/fpls.2022.907364)
Supplement: Supplementary file 1 [file Table_1.DOCX]

Supplementary Material

**Supplementary Table 1:** Primer sequences for Csa-miR395s

| Primer | Primer sequence（5ˊ-3΄） |
| --- | --- |
| U6 snRNA-F | GGAGGCAGAGGCATTGGA |
| U6 snRNA-R | CCCACCTTTGTTACCACCTTTG |
| Csa-miR395a-F | TGAAGTGTTTGGGGGAACTCT |
| Csa-miR395a-R | CAGTGCAGGGTCCGAGGTAT |
| Csa-miR395b-F | TGAAGTGTTTGGGGGAACTCC |
| Csa-miR395b-R | CAGTGCAGGGTCCGAGGTAT |
| Csa-miR395c-F | TGAAGTGTTTGGGGGAACTT |
| Csa-miR395c-R | CAGTGCAGGGTCCGAGGTAT |
| Csa-miR395d-F | CGGAGTTTCCCTGAATAACTTCATC |
| Csa-miR395d-R | CAGTGCAGGGTCCGAGGTAT |
